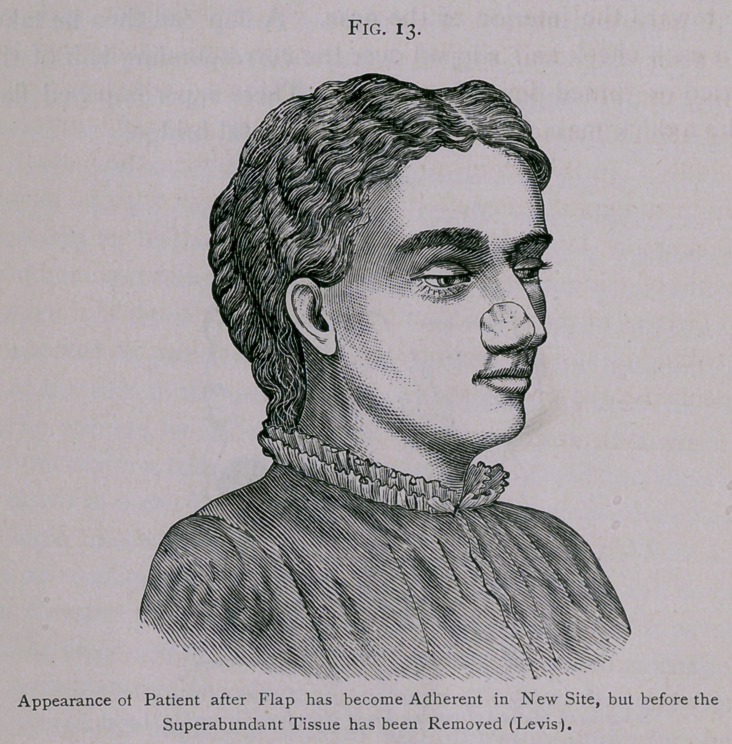# The Cure of Crooked and Otherwise Deformed Noses

**Published:** 1889-07

**Authors:** John B. Roberts

**Affiliations:** Professor of Anatomy and Surgery in the Philadelphia Polyclinic, Lecturer on Anatomy in the University of Pennsylvania, Surgeon to St. Agnes’ Hospital; 1118 Arch Street


Buffalo Medical and Surgical Journal

VOL. XXVIII. JULY, 1889. No. 12.

### Original Communications.

### *THE CURE OF CROOKED AND OTHERWISE DEFORMED NOSES.*

BY JOHN B. ROBERTS, A. M., M. D.,
Professor of Anatomy and Surgery in the Philadelphia Polyclinic, Lecturer on Anatomy in the University of Pennsylvania, Surgeon to St. Agnes' Hospital.

[Read in the Philadelphia County Medical Society, June 12, 1888.]

THERE are many instances of nasal deformity which are a great trial to those who have to bear the disfigurement through life, and whose correction would add much to the popular appreciation of surgery, and at the same time be a source of revenue to the operator whose skill succeeded in relieving the unsightly condition. Many of these defects are mere blemishes upon the face of the subject, rather than matters of very great importance. There may be little or no obstruction to breathing, and very little deformity to the eye of the observer, but the disfigurement causes such a mental effect on the patient as may even lead to a change in the disposition of the person. If we can relieve these minor disfigurements, and thereby relieve the worry and anxiety of the patient, we do something which is entirely justifiable.

Many forms of defect are found in the physical contour of the nose, and many are the causes giving rise to the varying types of nasal distortion. Faults of development are not uncommon. Accidental injuries, causing fracture or dislocation of the bones or cartilages, may occur in infancy, or at any time o life, and leave thereafter a very much disfigured organ. Blows of an opponent's fist, falls from a horse or carriage, and injuries received while playing ball, are not infrequently the disfiguring traumatism. We may also have deformity due to wounds made by cutting instruments, whereby portions of the soft structures have been lost, or to unseemly cicatrization after such wounds. Syphilis, giving rise to necrosis and ulceration of the bones and cartilages, thereby allowing the bridge of the nose to fall in, or other changes to take place, is a potent cause of nasal deformity. Ulceration of the alæ of the nose due to syphilis, while in the early stage necessitating active treatment, may later require surgical operation to reconstruct the lost part. Again, in epithelioma it may be necessary to cut away the ala and at once make a new structure. It may, at other times, be necessary to make a new tip to the nose after such an excision for malignant disease. By looking at a skull it is seen that if the septum and nasal

FIG. 1.

*a.* Saddle-back Nose. *b.* Tuberous Nose. *c.* Angular Nose.

bones are destroyed by syphilis, there is a great tendency for the bony arch, or bridge, to sink in and allow the tip of the nose to turn up; thus leaving only the small prominence formed by the wings and lobe of the nose, and causing great disfigurement of the front part of the face. Again, you may have lateral openings in the nose following specific ulceration. I have under observation at the present time a patient with an opening due to syphilitic ulceration, who has not yet agreed to an operation, but who will possibly do so in time.

I have drawn here a number of badly-shaped noses, which can be remedied by operation. We may have a nose that sinks in at the top, in which there is not much bridge. This is generally due to want of proper development of the bones and cartilages forming the septum, and is sometimes called the saddle-back nose (Fig. 1, *a*). A similar nose is often found in inherited syphilis. Then we may meet with a nose bent a good deal to one side (Fig. 2). A slight degree of this deformity is very common. Again, the organ may be not simply bent, but bent twice or irregularly twisted, as is shown in this drawing (Fig. 3). Bent and twisted noses are very often due to fractures

FIG. 2.

Bent Nose.

FIG. 3.

Twisted Nose.

received in early childhood. It is not unusual to see a nose with a large lump on the end of it, which may be due to hypertrophy of the lobe, or to a new growth developed there. This disfiguring condition is frequently due to acne rosacea, causing unusual development of tissue. This I call the tuberous nose (Fig. 1, *b*). I often see a man who has exceeding hypertrophy of the tip of his nose from acne rosacea, and whose son curiously has a similar nose, but not quite so greatly developed. Both these men could be greatly improved by operation. The last variety of which I shall here speak is what I call the angular nose (Fig. 1, *c*), because of the angular appearance of its dorsum, due to an unnatural bony prominence at the lower margin of the nasal bones. A lady came into my office a short time ago with such an elevation on the middle of the nose, due to a bony mass resulting from a fracture in infancy. There is also some stopping-up of the nostril from the fracture, and the occlusion has so interfered with respiration that she has been a mouth-breather since she was three years old. As a result of this, the lower jaw has not developed, and she cannot bring the incisor teeth together. She is much more concerned, however, about the appearance of her nose than about the obstruction to breathing. This patient, as well as those suffering from the other varieties of nasal deformity, can be greatly improved in appearance by proper and judicious surgical interference.

These illustrations, several of which are taken from a paper by Dr. S. B. Parsons, give an idea of the ordinary deformities which we have in the contour of the dorsum or bridge of the nose. A good many deformities also occur in the septum. In some of the cases just referred to there is not necessarily much deformity in the septum. In twisted and bent noses, however, there is usually some septal distortion, because the condition, as a rule, is the result of injury.

The peculiar broad and flattened appearance of the root of the nose seen in epicanthus, may be included in this enumeration of nasal deformities. Such cases are more apt, however, to be seen by those of us who practise ophthalmic surgery, than by general surgeons who do not attempt eye-surgery. The condition is remediable by dissecting an elliptical portion of skin, with its long diameter vertical, from the root of the nose and bringing the edges together with sutures. It is well in cases not very marked to delay operation, however, until the child has grown up, because the development of the bony structures of the nose may cause a spontaneous cure of the deformity.

In cases of crooked nose from traumatism, it is very common to have a greatly deformed septum. It is difficult, in fact, for a fracturing injury to cause much permanent nasal deformity without the cartilaginous or bony septum suffering some lesion at the time of the traumatism. The vomer and the perpendicular plate of the ethmoid are thin and easily broken, while the triangular cartilage is also readily fractured; hence, in nearly all deformed noses due to blows, deformity of the septum is found. The septal deviation usually interferes with respiration, because one nostril is more or less occluded. This causes change in the tone of voice, and induces other well-known symptoms.

Some persons are more annoyed by the nasal obstruction than by the want of physical comeliness in the external organ. Others care little about the patency of the nostrils, but worry greatly about the unsightly appearance of the deformed nose. Thus it is that patients come for the relief of one or the other condition, according to the character of their dispositions.

FIG. 4.

Normal. Curved. Angular. Enchondroma. Bridge of cartilage. Sigmoid.

Diagrams showing usual variations in shape of septum, as seen when observer looks into anterior nares.

Congenital deviation of the septum is by no means infrequent, and may in a similar manner interfere with proper respiratory performance. Such deviation of the septum is more apt to give rise to a curved bulging on one side than to an angular projection. Deviation of the septum due to injury is apt to be angular, and is very often accompanied by cartilaginous or bony outgrowths about the original lines of fracture. If the fracture has comminuted the septal structures to any great extent, both nostrils may become entirely filled with a mass of bone and cartilage. I once saw a case in which nostrils had to be made by actually quarrying through a mass of this kind by means of a chisel.

In these and other deformities small enchondromas may be found near the attachment of the septum to the floor of the nares, and in any form of septal distortion bridges of cartilage and bone may extend across the nasal chamber from the septum to the turbinated bone. These bridges at times are apparently formed by the coalescence of two opposing masses, just as a column is made in a cavern by the union of a stalactite with an opposing stalagmite. Cartilage tumors in the nose will often be found to have bony nuclei. This fact is worth remembering when the surgeon expects to attempt removal with a single incision made with a knife. Occasionally the septum has a double or sigmoid curve from above downward, as is shown in the last diagram on the blackboard. At other times the double curve is antero-posterior, so that one nostril is occluded in front and the other nostril occluded in the back of the nasal chamber. When one nostril is obstructed by what appears to be a deviated septum, it may happen that careful examination discloses the fact that there is no corresponding abnormal patency of the other nostril. The condition then is one of abnormal thickness of the septum, with a great degree of overgrowth on one side. This is to be treated by paring away the excess of cartilage or grinding away the redundant bone with the burr of the surgical engine.

In septal deviation the distortion is, fortunately, nearly always in the anterior two-thirds of the septum. Hence the deformity is more accessible to operative attack than would be the case if the converse were the rule.

There are many methods of relieving these nasal deformities. The method adopted must depend on the character of the condition. If the bridge is sunken, it must be raised and supported by the intra-nasal tissues. If the nasal bones are destroyed by syphilis, they must be substituted by new tissue, which is usually the tissue of the cheek or forehead, with possibly periosteal or osseous structures taken from the frontal region or from a lower animal. If there is a protuberance at the tip of the nose, it must be cut out and the two sides brought together so as to form a respectable-looking lobe. If the septum is bent over, it is necessary to open the occluded nostril, straighten the septum and keep it in the median line.

It is especially important that all recent fractures of the nose should be skilfully treated at once, in order to avoid subsequent deformity. This often is not done. There is a good deal of swelling and pain at the time of injury, which makes it difficult to determine the extent of the lesion; and as the condition is not serious, very little attention is given it, the structures are not accurately replaced, and in a few days, when the swelling subsides, the bones and cartilages are found to have united in bad positions. As a result, one of these deformed noses is the patient's burden through life, subjecting him to the criticism of his friends and the jeers of his enemies. Sometimes the displaced portion of bone is so sharp that it almost projects through the skin.

When a fracture occurs, diligent and intelligent efforts should at once be made to restore the bones to their proper position. Anesthesia should be resorted to if necessary, rather than have the replacement not absolutely correct. By looking at this specimen you see that the space under the nasal bones is not very great. In many text-books the surgeon is directed, in fractures of the nasal bones, to insert the end of a female catheter into the nostril, to push the bones into place, and then to put a plug or pad of lint in the nostril. Since the normal space under the nasal arch is narrow, and since in fracture there is great swelling of the mucous membrane, it is practically impossible to follow these directions, because the catheter is too wide. One can, however, get a thin instrument, like a steel director or the back of a small nasal saw, up under the bones. With this they can be elevated into place, and the bridge of the nose then moulded into proper shape with the fingers on the outside of the injured organ. These fractures heal with great rapidity; hence anything that is done must be done promptly, as in four or five days the union will be so firm that it will be almost impossible to restore the bones to their proper position, if the fragments have been left in a condition of deformity.

Syphilis of the nose is exceedingly liable to cause destruction of the bony and cartilaginous nasal structures, and, therefore, should be treated immediately on its recognition with *large* doses of mercury and potassium iodide. Many cases are, however, unrecognized, or are insufficiently treated, and as a result we have unsightly and unnecessary deformity. Superficial scars can sometimes be relieved by scraping away the irregularly cicatrized surface, since the irregularity in the cicatrization often looks worse than a large smooth scar would. A greater degree of damage will require for its repair the best efforts of a skilled plastic surgeon.

Just here let me say that in operating upon the skin of the nose or face, an incision made obliquely through the cutaneous tissue leaves a very faint cicatrix, because more correct apposition is obtained when the sutures are inserted. This is illustrated by cutting a card first obliquely and then perpendicularly to its surface. You see that when the pieces are brought together the line of union made at the former incision is not so distinct as that made at the latter place. A good deal of scarring may, however, be made about the nose, and yet not show very much finally, if the parts are brought accurately together, and the union takes place rapidly. This is especially true in the rough and coarse skin of many persons, where lines and creases about the nose are normally present.

There are some special plans of treatment which are valuable, and to which I shall now refer. When, after fracture of the nose and adjustment of the fragments, there is a tendency for them to become displaced again, they can usually be kept in position by what is known as Mason's method, though it is really a revival of an old method. With an ordinary awl or small drill, bore a hole transversely through the bones, and through this thrust a steel pin, to the projecting ends of which attach the ends of a rubber band, so placed as to pass across the bridge of the nose. This band should be so tightly stretched over the nose as to hold the bones up in position. The pin thus acts like the tie-beam which holds an arch together. The tendency of the rubber is to draw the pin up, and thus it supports the arch. I thought that

I had something new when on this same principle I put a pin through the nose and clamped a shot on the end; but I found that a similar thing had been used many years ago. When the surgeon does not wish to use the rubber band, he can clamp shot on the end of the pin, and have the upper part of the nose constantly under observation. Filling the nostrils with plugs of lint or other material to hold the parts in correct position is usually an ineffectual and uncomfortable method.

These methods may be employed in the treatment of recent fractures, or in cases where it is desirable to make a fracture in order to overcome deformity.

In correcting osseous deformity of the nose, whether congenital or due to fractures received many years previously, it is very easy with a proper chisel or saw to divide the bones to any extent and in any direction. For most of these operations the ordinary cold-chisel, previously ground to a fine sharp edge answers very well. These chisels can be bought for a few cents at the hardware stores. To loosen the nasal arch a small opening should be made at the side of the nose, at the junction of the nasal bone with the lateral cartilage, and the chisel then driven up between the nasal bone and the nasal process of the maxillary bone nearly as high, or quite as high, as the supraorbital ridge. The same thing should be done on the other side. The naso-frontal junction can be separated by introducing a strong steel instrument into the nose and elevating the nasal bones until fracture occurs near or at the suture line, or the bones can be cut loose by introducing a small chisel at the top of the nose. If it is considered necessary, small perforations with a drill may be made along the junction of the frontal and nasal bones before the fracture there is attempted. The bones can then be placed in any position desired and held there by a pin, thrust through the nose below them or made to perforate them after drilling with an awl. The circulation of the parts is so good that necrosis is almost impossible. The scars formed by the openings made for the introduction of the chisel cause much less deformity than that for which operation is performed. The nasal bones unite in four or five days without provisional callus, and if they are placed in good position a comely nose results. In some cases it may be better to introduce a small narrow saw into the nostril and saw the bone off on both sides. The chisel is rather a cruder way and perhaps not so accurate.

When the bridge of a nose is sunken in a good deal, when it is in fact a little like the saddle-back nose, a gutta-percha splint adjusted to the dorsum of the nose may be used to advantage, after a tenotome, introduced into the nostrils, has been used to cut all structures loose. The gutta-percha is placed in hot water until soft, and then moulded to the nose and allowed to cool, thus forming an external nasal splint. After loosening the bones and soft tissues, the operator carries a wire or silk suture through the nose below the splint and brings the ends up over the splint and ties them. This lifts the middle line or dorsum of the nose into the proper position. After a few days cicatrization takes place, and the normal conformation of the nose is maintained. I have not tried this method, but it seems as though it were a good one.

If there is an unseemly bony angle on the bridge of the nose, as in Fig. 1, *c*, it is proper to make a straight incision down the middle line of the nose, turn the skin aside, chisel off the mass, and then bring the skin together. There would be a red cicatrix for a short time, but it would soon lose its color and become a white line, which would be scarcely noticeable.

Another method of keeping the nose in place, is to put plugs of lint, oakum, wood, glass, gutta-percha, or silver, either solid or hollow, in the nostrils. They may be held in place by straps across the nostrils or by passing a pin through. If the plugs are tubular they soon become clogged, and should be removed every day or two to be cleansed.

When we come to the treatment of deviations of the septum we have to use different devices to meet different indications. Clamps have sometimes been applied so as to make pressure upon opposite sides of the septum. The clamp is left in position for several days, until the septum has become fixed in its new position. If the septum is bent to one side and the surgeon attempts to alter it by forcing it over and holding it in position by plugs, the patient will have to wear the plugs for weeks. Such patients may occasionally be seen going about the streets, and are noticeable because the string fastened to the plug is attached to the cheek by plaster. I do not believe that these simple measures will effect a cure except when the deviation is very slight. I have forced the septum over by the dilatation ot a laminaria uterine tent put into the nostril, but the result was very temporary. As soon as the plug is removed, there is a tendency for the resilient septum to resume its original position.

There are several other ways of overcoming septal deformity and the consequent nasal occlusion. One of the old methods is to cut out a piece of the septum at the point of deviation, if the deviation pertains to a limited area, so that breathing can go on

FIG. 5.

Roberts's Nasal Punch.

through either nostril. An objection to this method is that sometimes there occurs necrosis of the cartilage along the edge of the opening. At other times there is a constant tendency to the deposition of crusts thereon. I, however, do not think that the method is as objectionable as some have thought it. The operation is best done by the nasal-punch shown in the figure, which is a modification of that of Blandin, and acts better than his device, because it divides the mucous membrane on both sides of the septum more readily and perfectly.

Here is a pair of forceps, variously known as Steele's or Sajous' forceps, which cuts a stellate incision in the septum. By this means the septum is weakened, and can be pushed into place. If the deviation is very great, you may have to cut the septum in many places with these stellate forceps, and make it quite flexible. It then becomes almost as soft as wet cardboard, and can be pushed into position and be kept there by plugs or pins until cicatrization takes place.

The method of which I am most fond is to make a long incision at the most prominent portion of the deviation, and supplement this by chopping the septum full of incisions with the stellate punch. If some small triangular pieces are removed by the interlacing of the incisions made with the forceps, it makes no difference, since the openings left are very small and will soon become closed. If there is an angular deviation close to the palatal process of the superior maxillary bone, I make an incision from front to back at the most prominent part, and do not chop the upper portion with the stellate punch. If the deviation is a curved one, I split the cartilage along the most prominent portion and then chop the rest of the septum until it has lost its resiliency. Afterward I cut away with the chisel or saw any horizontal bony edge that may remain at the bottom. To hold the septum in place I usually use steel pins, either those with spherical heads of glass or the flat-headed pins which I devised some years ago. When the head of the pin is to be within the nostril, those with the glass heads are better; when the head is to lie flat against the exterior of the nose, the flat heads are preferable.

FIG. 6.

Roberts's Nasal Pin.

This method, which I employ so frequently to hold the septum in position, is a device of my own, and is difficult to render clear by a mere description. After having divided the septum, with a knife, along the most prominent part of the deviation, and having made the cartilage flexible by multiple incision with the stellate punch, I introduce a pin into the more open nostril and thrust its point through the anterior part of that portion of the septum which I wish to control and keep in a new relation to the other portions. After I have displaced this part into the desired position, I thrust the point of the pin onward and bury its point deep in the tissues at the back part of the nasal chamber which was formally occluded. This holds the septum firmly in its new position. The head of this pin will be just inside of the anterior naris which was not obstructed, and will lie against the columella. It should be allowed to remain

FIG. 7.

Roberts's method of holding septum in correct position by means of pins.

The upper part of the septum, immediately above the oblique incision, projected too far this way (*i. e.*, toward the reader). It is now pressed the other way (*i. e.*, from the reader), and is held there by the pins.

about one week, for, if left a longer time, its head will probably cause ulceration of the columella, and may become deeply buried in the tissues of the columella. Its work is usually accomplished within a week. It is often well to introduce a second pin from the external surface of the front of the nose just below the nasal bones, which aids in keeping the septal cartilage pinned into proper place. If this pin has a flat head, it may be covered with a small square of court-plaster. The patient can then go about the streets without attracting attention.

By this method there is no plug to obstruct the nostril, and the patient can snuff up any cleansing wash desired. It is the neatest and most satisfactory way of rectifying deviated septa which has ever been employed by me, or with which I am acquainted. Its employment for four or five years has given me increased confidence in its value, both in cases of simple deviation of the septum, and in cases where more complicated operations have been simultaneously required to correct nasal distortion.

Some operators correct crooked septa and other deviations of the nose by seizing the septum between the two blades of an Adams's forceps, and fracturing the intra-nasal structures sufficiently to allow of their reposition in conformity with the natural outlines. A head-band with pads and screws, a mask with adjustable pads to produce pressure on various portions of the nose, or an intra-nasal truss or clamp, is then called into requisition to maintain the position so obtained. This mode of operating seems to me less accurate, less scientific, and much more annoying to the patient than my method by incision and pins.

It is said that in some instances of deviated septum, the middle turbinated bone in the non-occluded nasal cavity is greatly hypertrophied, and may, by reason of its bulk, interfere with the surgeon's efforts to push the septum into the median line. If such interference exists, the turbinated bone should be partially or entirely excised, in order that reposition of the septum may be effected.

In operations which are not very severe, local anesthesia is readily obtained by placing a pledget of cotton, moistened with a four per cent. solution of cocaine, in the nostril and allowing it to remain for a few minutes. In operations which are prolonged, it is better to give ether. The disadvantages of anesthesia are that the blood runs into the throat and the patient vomits; but, on the other hand, the surgeon is more apt to do a thorough and satisfactory operation if he has an anesthetized person with which to deal. I do not like to plug the posterior nostrils, because the string coming out through the anterior nares may be cut during the operation and the plugs be thus allowed to fall into the pharynx. Occasional cleaning of the fauces, by turning the patient on his face, is all that is required when no plugs are used.

In order to get rid of horizontal ridges of bone and cartilaginous growths that may be present in either nostril, I use one of these small saws, or a chisel and mallet. If the growth is entirely cartilaginous, it may be removed with a bistoury or with this sickle-shaped cartilage knife, which is placed behind the growth and drawn forward.

The enchondromatous tumors found growing from the septum often have a bony center, and the surgeon may find it difficult to remove it with the ordinary knife, and be obliged to use a chisel or a saw. There is a little knack required in removing these cartilaginous masses neatly. If you undertake to use a knife, or a knife and saw, in the ordinary way, you readily cut through the mucous membrane on the upper surface of the tumor, and the tumor itself, but when you come to the mucous membrane underneath, which is very loose and flaccid, it does not offer sufficient resistance to be divided quickly and neatly. The tumor is perfectly detached above, but is attached below by a band of mucous membrane which is difficult to divide, because you cannot see where to apply the forceps or scissors on account of the blood in the nostril. To avoid this annoyance, it is best to cut through the mucous membrane below the tumor first, and afterward to cut down from above with the saw, chisel, or knife, when the tumor at once falls out. Another device that is very satisfactory is to first transfix the tumor with a barbed needle, which acts as a handle by which to hold the tumor steady while excising it. Of course, this is not available if the enchondroma has much bone in its interior.

FIG. 8.

Roberts's Barbed Needle.

Dr. Seiler has suggested that a grooved director be slipped under the cartilaginous ridge or tumor, with its groove presenting toward the mass to be removed, and that a triangular or plough-shaped knife be then pushed along the groove so as to cut off the excrescence. He also employs, for removing such growths, chisels and gouges of varying shapes set at an angle in a detachable handle. These devices facilitate at times the neatness and rapidity of the operation.

The means to be employed in correcting the deformity in cases of bent and twisted nose vary with the characteristics of each case. Indeed, it often happens that the nasal distortion combines the peculiarities of more than one type of deformity. I recently saw a gentleman, for example, whose badly-bent nose, due to injury sustained in a runaway accident, was made additionally ugly by a large and unshapely lobe or tip. It is necessary, as a rule, in remedying bent or twisted noses, to cut the cutaneous structures thoroughly loose from the septum and nasal arch by free subcutaneous incisions. This is done with a tenotome introduced usually, but not always, through the nostril, and then carried under the skin. I prefer this method, in order to make as few punctures on the cutaneous aspect of the nose as possible; though, as before stated, small incisions or punctures leave in the end very little scarring. After the external nose has been thus loosened by subcutaneous "undermining" with the tenotome, it is necessary to get rid of bony projections by means of the chisel, to cut away any bone or cartilage occluding the nostril, to divide and readjust the distorted septum which is usually present, and then to seize the nose with the fingers and twist or bend it into its normal position. When the desired position has been obtained, the parts must be fixed there by pins or other means until union has occurred. This requires about a week's time.

The errors likely to be committed are insufficient division of the distorted structures, and the application of too little force when the attempt is being made to press the organ back into its normal relation with the face. If the surgeon will recollect what a great degree of force is required to produce the distortion found in accidental nasal deformity, he will better appreciate how much force he may use with impunity in endeavoring to correct these and similar disfigurements.

The tuberous nose and the angular nose are easily improved by simply cutting away the excess of tissue and uniting the resulting wound with fine sutures of catgut or silk. In these, as in all plastic operations, the ingenuity of the surgeon must be exercised to give the least scarring and the most perfect contour. The same shaped nose should not be repaired in exactly the same way in every sort of face. The peculiar facial lines of the individual or the shape of his other features has a good deal to do with determining the variety of operative procedure best adapted to the requirements. The question involves not only manual dexterity on the part of the operator, but a considerable degree of artistic training. This is equally true when portions of the nose have to be constructed from the cheeks, lips, or finger-tip. Such rhinoplastic operations, however, are rather beyond the scope of the present paper.

FIG. 9.

Diagram of Original Deformity.

FIG. 10.

Diagram of Flap.

FIG. 11.

Diagram of Sutures.

There is only one thing more of which to speak. It is the correction of those great deformities which occur from sinking of the whole upper portion of the nose as the result of syphilis. A woman came to my office a short time ago, who was in the habit of wearing continually a thick black veil to hide a deformity due to such a loss of the cartilaginous nasal bridge. The point of the nose was turned up, and there was a deep groove over the area of the sunken nasal bridge. What is required in these cases is the construction of a new bridge. This is difficult where there are no remains of the original bony structures. I accomplished it pretty well here, however, as the nasal bones remained. I first made a transverse incision across the nose in the deep groove at the line of junction of the deformed organ and the face, cutting thereby directly into the nasal chambers, and then pared everything loose inside. The nose was then only attached to the face by the columna and the alæ, and could be pulled downward and forward so as to give its tip a natural prominence or elevation beyond the cheeks. This procedure left a large opening between the lower portion of the nose and the nasal arch and frontal bone, through which I could look directly into the nasal chambers; and which had to be covered with a flap. I laid over it a triangular flap of skin and superficial fascia, dissected from the space between the eyes and from the forehead. This was slipped downward and sutured in position. The raw surface left on the forehead and at the root of the nose was drawn together as much as possible by sutures, or allowed to heal by granulation. I thought it better to make a triangular incision on the forehead and slip this flap down to cover the opening below, than to turn in cheek flaps. The latter method would have caused more scarring. The soft parts so transplanted were held in place by sutures, and somewhat elevated like a normal nasal bridge by pins thrust across the nose from side to side, upon the points of which perforated shot were threaded and clamped. Pieces of rubber drainage-tube were thrust through the nostrils and upwards under the new bridge, to give greater support. These were not introduced to facilitate respiration, as I intended the patient to breathe entirely through her mouth. When the wound had healed, the patient had a nose which was of a pretty good shape, but it was somewhat flabby in the region of the bridge. This is overcome by wearing a pair of spectacles which have little pads on each side of the nose like eyeglasses. These pads pinch up the soft bridge and give it a more natural appearance. This patient has a much better nose than before, although it is, of course, not as good as a normal nose. The other day she came into my office wearing a thin veil instead of the thick black one she had been accustomed to wear. This proved to me that she considered the deformity to have been greatly overcome.

Another way of relieving this sort of deformity is to take a large oval flap from the forehead and turn it down with the skin surface toward the interior of the nose. A flap can then be taken from each cheek and slipped over the corresponding half of this everted or turned-down frontal flap. These super-imposed flaps make a thick mass which simulates the nasal bridge.

FIG. 12.

Diagram of Method of making Ala of Nose from Upper Lip (Levis).

Portions of periosteum and thin plates of bone may perhaps be chiseled from the forehead or superciliary ridges and successfully turned into the gap to give solidity to such newly constructed bridges; but I do not know how successfully such operations have terminated, if, indeed, they have ever been actually undertaken. Bone-grafts from lower animals may prove valuable. If there is absence of the ala as the result of ulceration or the removal of tumor, the surgeon should turn in a portion of the cheek or lip. In these plastic operations he can often bring together the edges of the wound left by the transfer of tissue, so as to leave a mere linear scar. Under-cutting the skin before putting in the sutures permits the elastic skin to be drawn over the gap.

If the columella is gone, a portion of the upper lip, including its entire thickness, can be taken out of the center of the lip and turned up to make a columna. It is then necessary to bring the

FIG. 13.

Appearance of Patient after Flap has become Adherent in New Site, but before the Superabundant Tissue has been Removed (Levis).

divided upper lip together as in hair-lip. This manœuvre lessens the size of the lip, but that is rather an advantage, since in such nasal cases the lip usually appears to be relatively too large.

You can see from what I have said that many persons having great deformity of the nose can be much relieved if you study the artistic bearings of the case. It is often only necessary to diminish what is too big in order to restore the proper relationship of the parts. If the bridge of the nose is too small, the surgeon can often give the nose a symmetrical appearance by getting rid of a portion of the tip which is relatively, though perhaps not actually, too large. When there is a disparity between the nose and the lips or chin, he may be able to alter the size of the tip of the nose, if he cannot change the other features. The operation which leaves the least scar and brings the parts in the best mutual relation, as to size and appearance, is that which should always be selected.

I have endeavored to show in this address the erroneous character of the teaching which advocates letting deformed noses alone, and which recommends surgeons to advise their patients to bear the affliction of an ugly nose with becoming grace and humility. In this, as in all surgical proceedings, the patient has a right to expect every effort consistent with safety to be made by the surgeon. Procedures such as I have described are practically devoid of danger, are efficient in relieving the mental and physical distress of the patient, and should be done when the patient is willing to assume the moderate temporary inconvenience coincident with the operation.

1118 Arch Street.

## Figures and Tables

**Fig. 1. f1:**
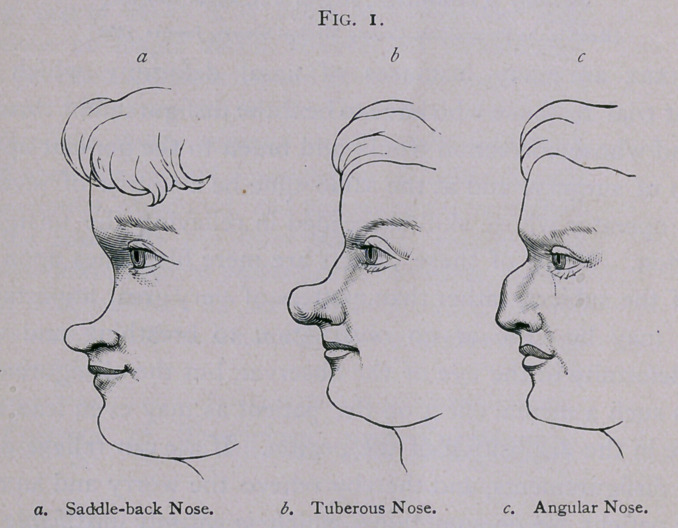


**Fig. 2. f2:**
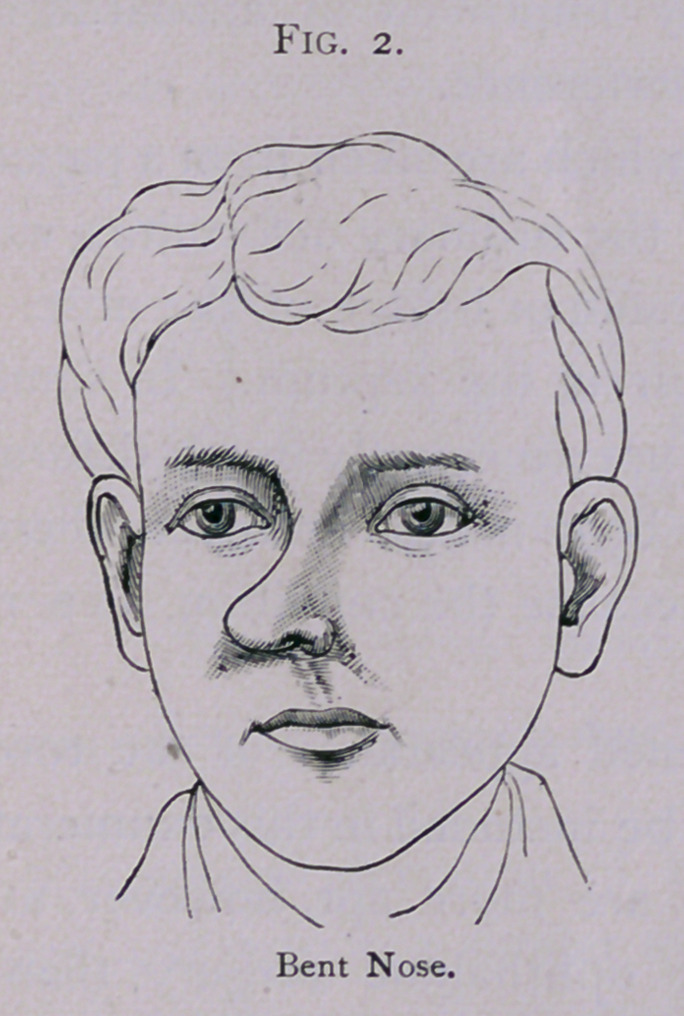


**Fig. 3. f3:**
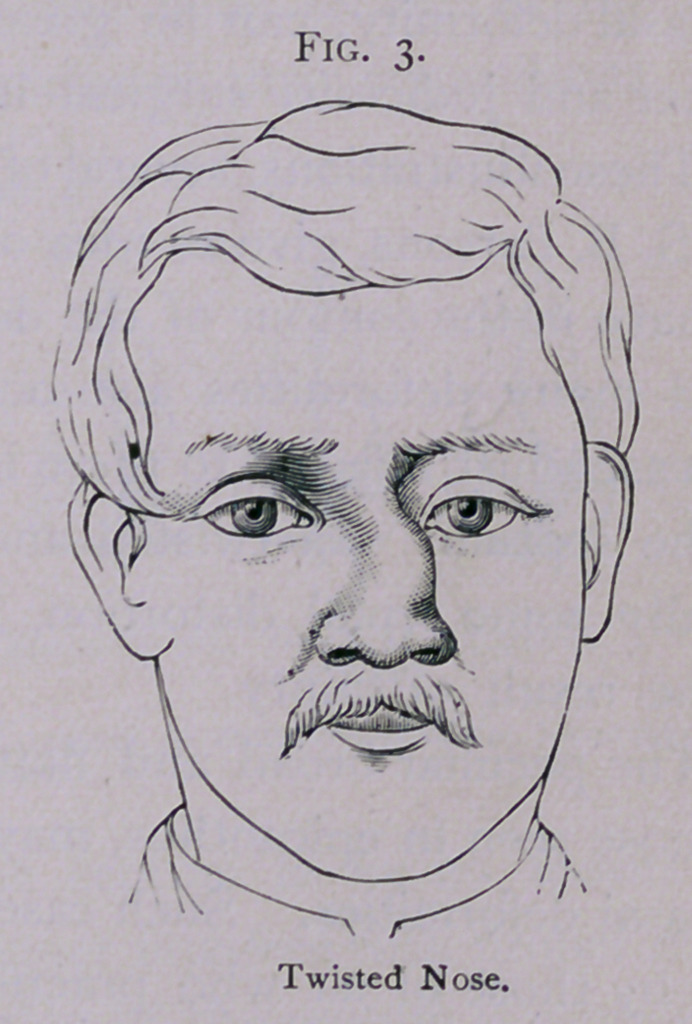


**Fig. 4. f4:**
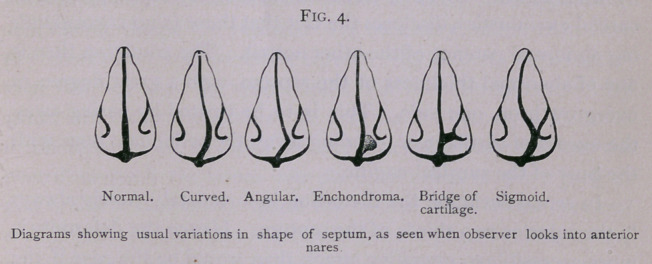


**Fig. 5. f5:**
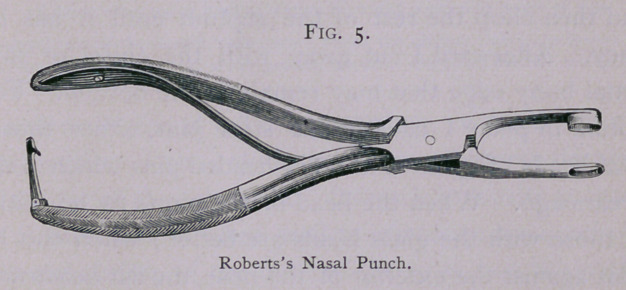


**Fig. 6. f6:**
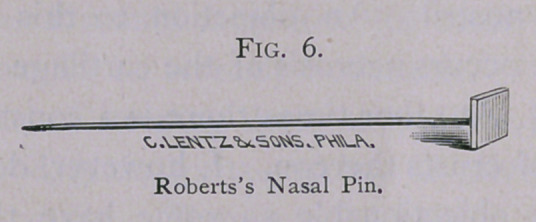


**Fig. 7. f7:**
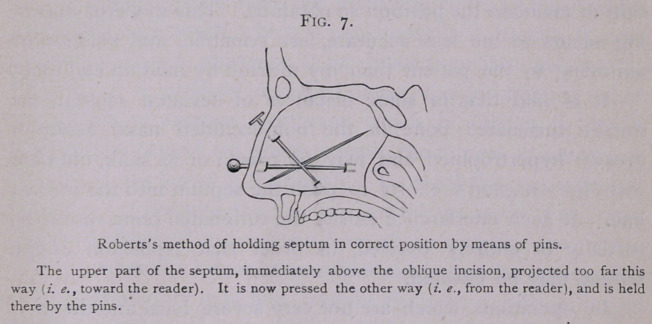


**Fig. 8. f8:**
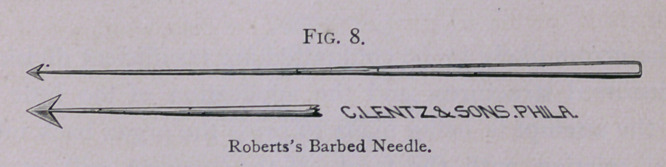


**Fig. 9. f9:**
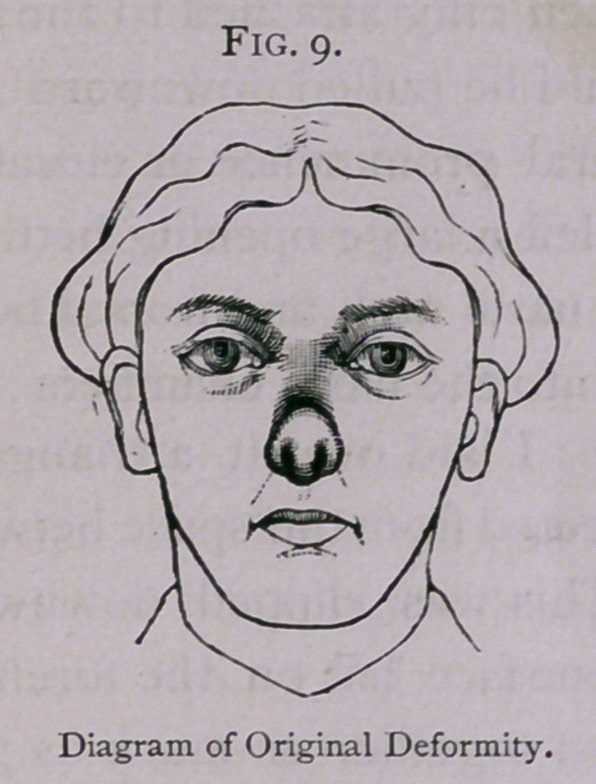


**Fig. 10. f10:**
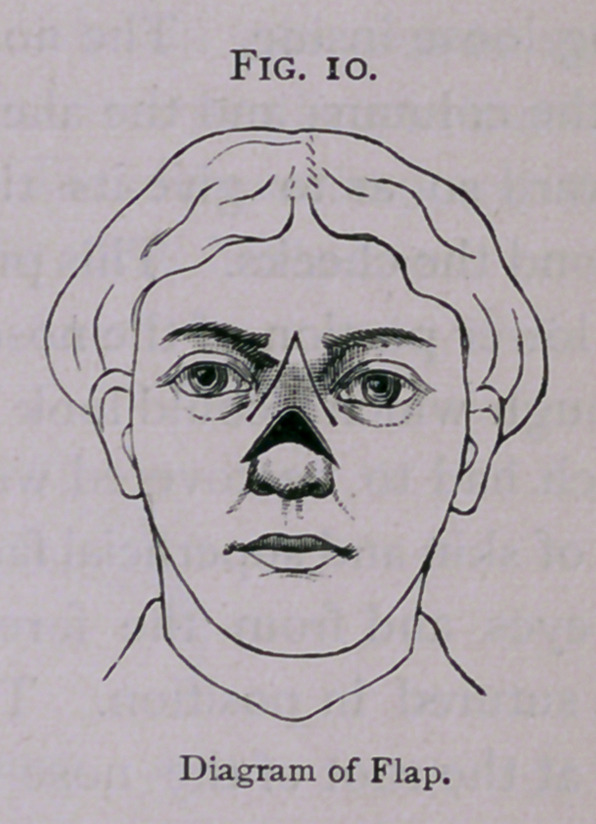


**Fig. 11. f11:**
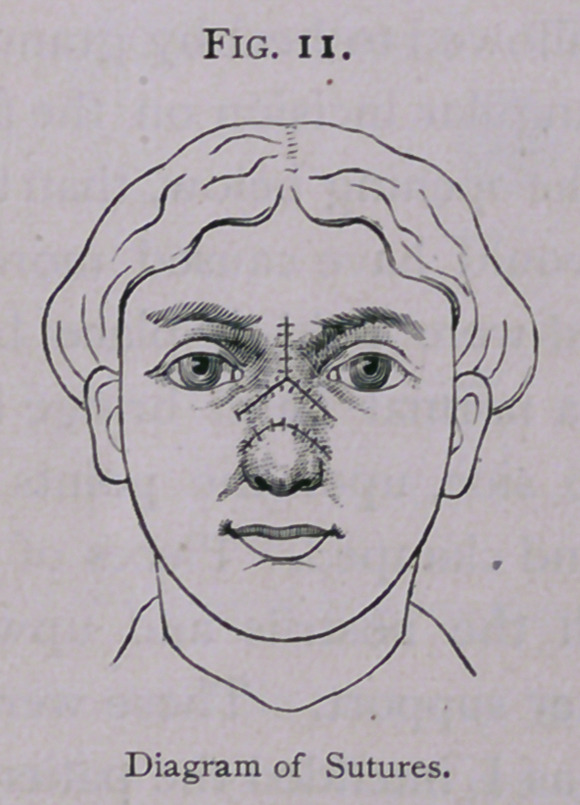


**Fig. 12. f12:**
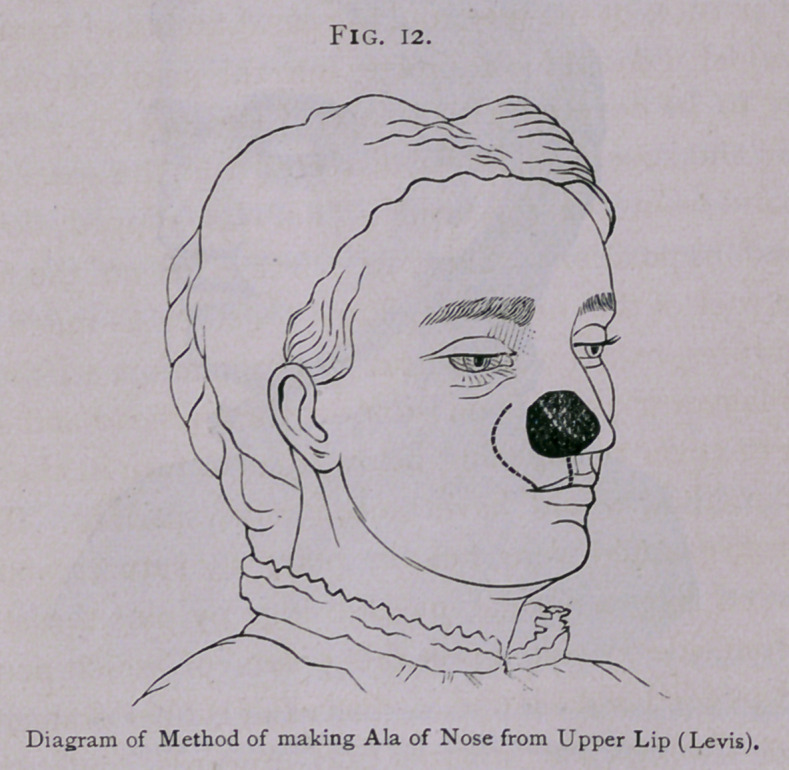


**Fig. 13. f13:**